# The impact of receptor of advanced glycation end‐products polymorphisms on prostate cancer progression and clinicopathological characteristics

**DOI:** 10.1111/jcmm.17025

**Published:** 2021-10-27

**Authors:** Ying‐Erh Chou, Ming‐Ju Hsieh, Shian‐Shiang Wang, Chia‐Yen Lin, Yen‐Yu Chen, Yung‐Chuan Ho, Shun‐Fa Yang

**Affiliations:** ^1^ School of Medicine Chung Shan Medical University Taichung Taiwan; ^2^ Institute of Medicine Chung Shan Medical University Taichung Taiwan; ^3^ Department of Medical Research Chung Shan Medical University Hospital Taichung Taiwan; ^4^ Oral Cancer Research Center Changhua Christian Hospital Changhua Taiwan; ^5^ College of Medicine National Chung Hsing University Taichung Taiwan; ^6^ Graduate Institute of Biomedical Sciences China Medical University Taichung Taiwan; ^7^ Division of Urology Department of Surgery Taichung Veterans General Hospital Taichung Taiwan; ^8^ Department of Applied Chemistry National Chi Nan University Nantou Taiwan; ^9^ Department of Medical Applied Chemistry Chung Shan Medical University Taichung Taiwan

**Keywords:** polymorphism, prostate cancer, RAGE

## Abstract

The receptor for advanced glycation end products (RAGE) overexpression was suggested to be associated with prostate cancer development and poor prognosis. In this study, we focused on the correlations between the clinicopathological characteristics and susceptibility of prostate cancer and *RAGE* single‐nucleotide polymorphisms (SNPs). In 579 prostate cancer patients, the *RAGE* SNPs rs1800625, rs1800624, rs2070600 and rs184003 in patients with or without grade group upgrade were analysed with real‐time polymerase chain reaction. The results demonstrated that the prostate cancer patients who carried the *RAGE* SNPs rs2070600 ‘GA’ genotypic variants were significantly associated with lower risk to develop grade group upgrade. Moreover, patients with the *RAGE* rs1800625 ‘TC + CC’ genotypic variants were associated with higher risk of perineural invasion. In 343 prostate cancer patients who carried the *RAGE* rs1800625 ‘TC + CC’ genotype without grade group upgrade were correlated with higher risk of biochemical recurrence and perineural invasion. In the analysis of TCGA database, significant differences of the RAGE mRNA level were found between the normal controls and prostate cancer patients (*p* < 0.0001), and the pathologic stage N1 and N0 patients (*p* = 0.0027). The prostate cancer patients with high RAGE expression were associated with lower overall survival rate (*p* = 0.025). In conclusion, our results have revealed that the *RAGE* SNPs rs2070600 and rs1800625 were associated with the grade group upgrade of prostate cancer and clinical status. The *RAGE* polymorphisms may provide as a pivotal predictor to evaluate prostate cancer disease progression and prognosis.

## 1 INTRODUCTION

Prostate cancer (PCa) is a global health problem with considerable diversity in epidemiology and genomics.^1^ In Taiwan, prostate cancer is the fifth most prevalent cancer and ranks the seventh highest cancer‐related mortality rate.^2^, ^3^, ^4^ Epidemiological risk factors such as ageing and high fat consuming diet were suggested to raise the incidence of PCa in Taiwan.^2^, ^5^ RAGE, or the AGER, is the receptor for advanced glycation end products (AGEs).^6^, ^7^ The AGEs are non‐enzymatic protein modifications, which were produced during ageing.^7^, ^8^ In prostate cancer, overexpression of RAGE and its ligand amphoterin were found to be correlated with tumour development and poor prognosis.^9^, ^10^, ^11^ The RAGE expression was observed to be correlated with apoptosis induction and inhibition of prostate tumour growth,^12^ and the RAGE quantification of human prostate cancer samples has been confirmed that increased uptake of RAGE was corresponding to increasing of Gleason scoring.^13^

The polymorphisms of *RAGE* were suggested to be associated with various cancers,^14^, ^15^, ^16^ including oral cancer,^17^ breast cancer,^18^, ^19^, ^20^, ^21^, ^22^, ^23^, ^24^ lung cancer,^25^, ^26^, ^27^, ^28^ gastric cancer,^29^, ^30^, ^31^ hepatocellular carcinoma (HCC),^32^ pancreas cancer,^33^ cervical cancer,^34^, ^35^ urothelial cell carcinoma^36^ and colorectal cancer.^37^ Previous studies revealed that the *RAGE* rs1800625 polymorphism was correlated with the increasing of cancer risk in various cancers including oral cancer and gastric cancer.^15^, ^17^, ^29^ Moreover, the ‘TT’ polymorphisms of rs184003 were suggested to be correlated with poorer disease‐specific survival on urothelial cell carcinoma,^36^ and individuals who carried the rs184003 T allele were found to exhibit increased risk of breast cancer.^20^ However, the *RAGE* polymorphisms to prostate cancer progression and clinicopathologic characteristics remained not well‐investigated. In this study, we focused on four SNPs of *RAGE* rs1800625, rs1800624, rs2070600 and rs184003, and try to elucidate their correlations to clinicopathologic characteristics and susceptibility of prostate cancer.

## 2 MATERIALS AND METHODS

### 2.1 Study subjects

In the current study, 579 prostate cancer patients with adenocarcinoma were enrolled as the study group. During 2012–2017, the patients who involved in our study have received robotic assisted radical prostatectomy at Taichung Veteran General Hospital. The informed consent was confirmed and acquired from each individual who enrolled in our study (IRB No. CE19062A). The medical information including the age at diagnosis (years), initial PSA level at diagnosis (ng/ml), clinical and pathological TNM staging, pathologic Gleason grade group, perineural invasion, seminal vesicle invasion, lymphovascular invasion, biochemical recurrence and D'Amico classification was acquired from the personal medical records for each patient.^38^ Before this study started to initiate, the certification and approval was confirmed by the Institutional Review Broad (IRB) of the Taichung Veteran General Hospital.

### 2.2 Sample preparation and DNA extraction

For genomic DNA extraction, the peripheral blood specimens from normal controls and prostate cancer patients who enrolled in our study were collected. The samples of peripheral whole blood were preserved in EDTA containing tubes and centrifuged with the settings of 3000 *g* for 10 min. The buffy coats extracted from centrifuged whole blood specimens were further applied for the DNA extraction.^39^ The Tris‐EDTA (TE) buffer was used to dissolve DNA and to complete the DNA elution. The final extracted DNA was prepared as DNA template in polymerase chain reactions (PCRs).^40^

### 2.3 Selection of *RAGE* SNPs and *RAGE* SNPs genotyping

In our current study, a total of four SNPs of *RAGE* rs1800625, rs1800624, rs2070600 and rs184003 were selected from the International HapMap Project database.^41^ The *RAGE* rs1800624 polymorphism was suggested to contribute to increase breast cancer and lung cancer risk.^23^, ^42^ The *RAGE* rs2070600 polymorphism was associated with significant breast cancer and gastric cancer risk.^20^, ^30^ The assessment of allelic discrimination for the *RAGE* rs184003, rs2070600, rs1800624 and rs1800625 SNP was performed with ABI StepOne Software v2.3 Real‐Time PCR System. The genotyping was analysed with the TaqMan assay. The SDS 7000 series software (Applied Biosystems) was applied for the analysis and calculation of the final data of genotyping.

### 2.4 Statistical analysis

To compare the age at diagnosis (years), PSA at diagnosis (ng/ml), clinical T stage, pathologic T stage, pathologic Gleason grade group, pathologic N stage, perineural invasion, seminal vesicle invasion, lymphovascular invasion, biochemical recurrence and D'Amico classification between the patients with or without grade group upgrade, Student's t test and chi‐squared test or was used between these two groups. A statistical significant was considered if *p* < 0.05 presents. To evaluate the odds ratio (OR) with their 95% confidence intervals (CIs) of the association between the prostate cancer risk and the clinical pathological characteristics and genotypic frequencies, logistic regression models were adopted for data analysis and assessment. The analysis of all the data in our study was evaluated and calculated with SAS statistical software (Version 9.1, 2005; SAS Institute).

## 3 RESULTS

In 579 patients with prostate cancer, the distribution of demographical characteristics was demonstrated in Table 1. In our study, we found that the distributions of age at diagnosis (years) >65 of the patients with no grade group upgrade were 58.6% (201/343) and 56.4% (133/236) of the patients with grade group upgrade. The PSA at diagnosis >10 ng/ml between these two groups was 54.5% (187/343) and 51.7% (122/236), respectively. A statistical significant difference was found for clinical T stage (*p* = 0.002), pathologic N stage (*p* = 0.034) and D’Amico classification (*p* = 0.001) between the prostate cancer patients with or without grade group upgrade (Table 1).

**TABLE 1:** The distributions of demographical characteristics in 579 patients with prostate cancer

Variable	Grade group upgrade		*p* Value
	No (*n* = 343)	Yes (*n* = 236)	
Age at diagnosis (years)			
<65	142 (41.4%)	103 (43.6%)	*p *= 0.591
>65	201 (58.6%)	133 (56.4%)	
PSA at diagnosis (ng/ml)			
≤10	156 (45.5%)	114 (48.3%)	*p *= 0.503
>10	187 (54.5%)	122 (51.7%)	
Pathologic Gleason grade group			
1 + 2 + 3	295 (86.0%)	189 (80.1%)	*p *= 0.059
4 + 5	48 (14.0%)	47 (19.9%)	
Clinical T stage			
1 + 2	284 (82.8%)	217 (91.9%)	*p *= 0.002*
3 + 4	59 (17.2%)	19 (8.1%)	
Pathologic T stage			
2	190 (55.4%)	116 (49.2%)	*p *= 0.139
3 + 4	153 (44.6%)	120 (50.8%)	
Pathologic N stage			
N0	307 (89.5%)	223 (94.5%)	*p *= 0.034*
N1	36 (10.5%)	13 (5.5%)	
Seminal vesicle invasion			
No	267 (77.8%)	185 (78.4%)	*p *= 0.876
Yes	76 (22.2%)	51 (21.6%)	
Perineural invasion			
No	95 (27.7%)	60 (25.4%)	*p *= 0.544
Yes	248 (72.3%)	176 (74.6%)	
Lymphovascular invasion			
No	284 (82.8%)	198 (83.9%)	*p *= 0.139
Yes	59 (17.2%)	38 (16.1%)	
D’Amico classification			
Low risk	46 (13.4%)	14 (5.9%)	*p *= 0.001*
Intermediate risk	113 (32.9%)	107 (45.3%)	
High risk	184 (53.7%)	115 (48.7%)	
Biochemical recurrence			
No	236 (68.8%)	168 (71.2%)	*p *= 0.540
Yes	107 (31.2%)	68 (28.8%)	

The distribution frequency of *RAGE* genotypes of 579 prostate cancer patients was listed in Table 2. The highest distribution frequencies in prostate cancer patients of *RAGE* polymorphisms rs1800625, rs1800624, rs2070600 and rs184003 were homozygous for TT, homozygous for TT, homozygous for GG and homozygous for GG, respectively. The odds ratios (ORs) and their 95% confidence intervals (CIs) were evaluated by logistic regression models. After adjustment for the effects of age at diagnosis, PSA levels at diagnosis, clinical T stage, pathologic T stage, pathologic N stage, pathologic Gleason grade group, perineural invasion, seminal vesicle invasion, lymphovascular invasion, biochemical recurrence and D’Amico classification, a significant difference (*p* = 0.019) and adjusted odds ratios (AORs) = 0.628 with CIs = 0.426–0.926 was observed in prostate cancer patients with or without grade group upgrade with *RAGE* rs2070600 ‘GA’ genotype compared with the wild‐type (WT) ‘GG’ carriers (Table 2).

**TABLE 2:** Distribution frequency of *RAGE* genotypes in 579 patients with prostate cancer

Variable	Grade group upgrade		AOR (95% CI)	*p* Value
	No (*n* = 343)	Yes (*n* = 236)		
rs1800625				
TT	287 (83.7%)	194 (82.2%)	1.00	
TC	54 (15.7%)	37 (15.7%)	0.990 (0.614–1.598)	0.968
CC	2 (0.6%)	5 (2.1%)	2.652 (0.495–14.210)	0.255
TC + CC	56 (16.3%)	42 (17.8%)	1.062 (0.669–1.685)	0.800
rs1800624				
TT	259 (75.5%)	181 (76.7%)	1.00	
TA	75 (21.9%)	48 (20.3%)	0.932 (0.604–1.437)	0.750
AA	9 (2.6%)	7 (3.0%)	1.003 (0.352–2.864)	0.995
TT + AA	84 (24.5%)	55 (23.3%)	0.940 (0.622–1.420)	0.770
rs2070600				
GG	208 (60.6%)	159 (67.4%)	1.00	
GA	120 (35.0%)	60 (25.4%)	0.628 (0.426–0.926)	0.019*
AA	15 (4.4%)	17 (7.2%)	1.452 (0.673–3.133)	0.341
GA + AA	135 (39.4%)	77 (32.6%)	0.716 (0.497–1.030)	0.072
rs184003				
GG	230 (67.1%)	163 (69.1%)	1.00	
GT	104 (30.3%)	66 (28.0%)	0.902 (0.613–1.328)	0.602
TT	9 (2.6%)	7 (2.9%)	1.081 (0.366–3.190)	0.888
GT + TT	113 (32.9%)	73 (30.9%)	0.916 (0.629–1.333)	0.646

Note

The odds ratios (ORs) and with their 95% confidence intervals (CIs) were estimated by logistic regression models.

* *p* Value < 0.05 as statistically significant.

**FIGURE 1:**
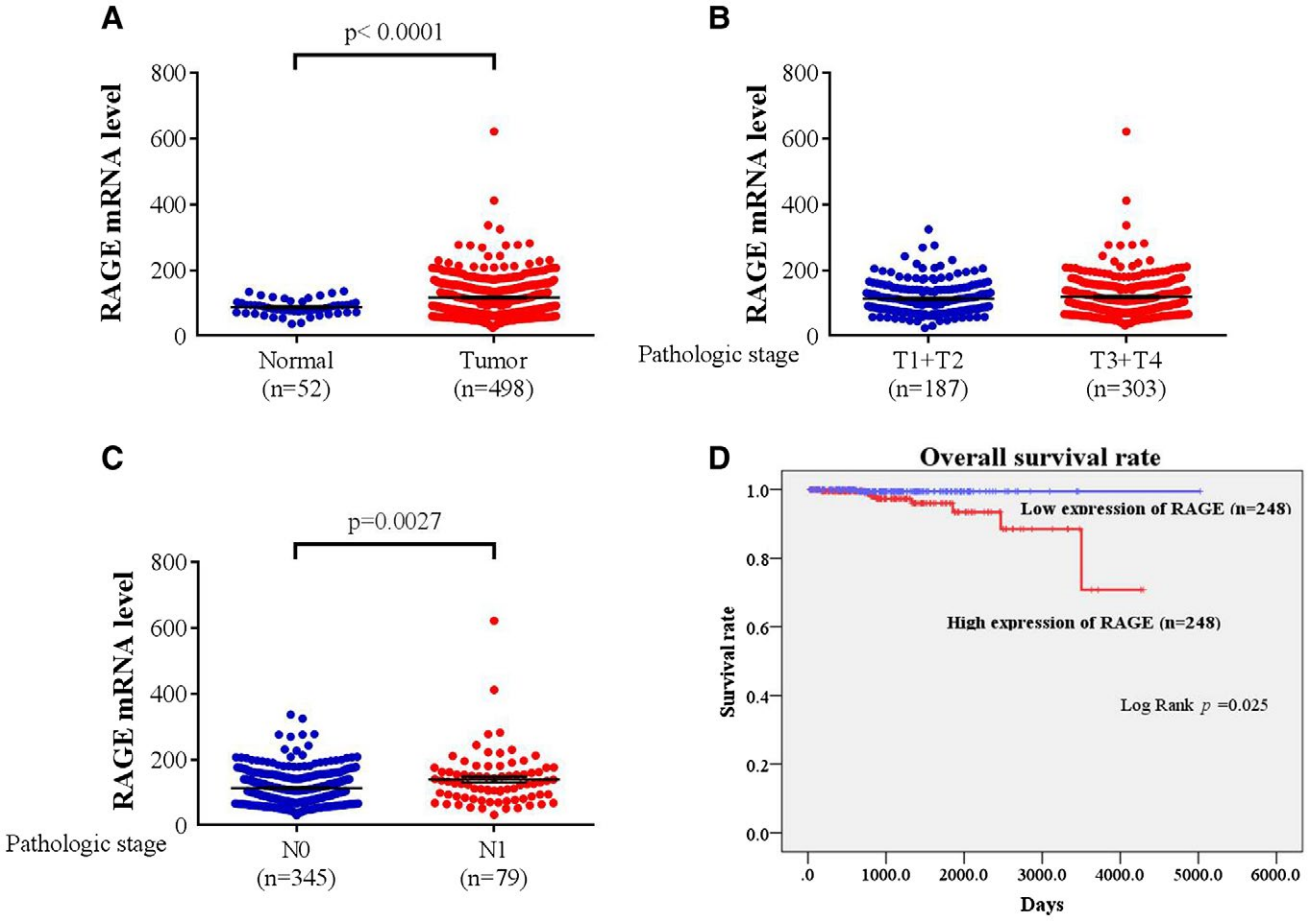
RAGE mRNA level of prostate cancer patients from TCGA database. (A) RAGE levels were compared between the prostate cancer tumour tissues and normal tissue. (B) RAGE levels were compared between the pathologic T1 + T2 stage and T3 + T4 stage. (C) RAGE levels were compared between the pathologic N0 stage and N1 stage. (D) Analysis of overall survival and RAGE mRNA expression in prostate cancer patients from TCGA database. The overall survival curve was produced for overall prostate cancer patients (*n* = 248). The effect of RAGE mRNA expression on the overall survival of prostate cancer patients was evaluated by Kaplan–Meier method. The *p* values were determined with log‐rank test. RAGE, receptor of advanced glycation end‐products; TCGA, The Cancer Genome Atlas

We further analysed the distribution frequency of *RAGE* genotype in 270 patients with prostate cancer with PSA ≤ 10. Statistical significant differences were found in patients who carried the *RAGE* rs2070600 ‘GA’ (AOR = 0.304, 95% CI = 0.164–0.563; *p* < 0.001) and ‘GA + AA’ (AOR = 0.375, 95 CI = 0.214–0.657; *p* = 0.001) genotype (Table 3). To clarify the role of *RAGE* genetic polymorphisms in prostate cancer progression, we analysed the clinical status and *RAGE* genotypic frequencies in 579 prostate cancer patients. The *RAGE* rs1800625 ‘TC + CC’ genotype was found to be significantly associated with higher risk of perineural invasion (OR = 2.272, 95% CI = 1.267–4.074; *p* = 0.005) (Table 4). We further analysed the clinical status and *RAGE* rs1800625 genotypic frequencies in 343 patients with no grade group upgrade. The *RAGE* rs1800625 ‘TC + CC’ genotype was significantly associated with perineural invasion (OR = 2.610, 95% CI = 1.185–5.749; *p* = 0.014) and biochemical recurrence (OR = 1.843, 95% CI = 1.024–3.317; *p* = 0.039) in patients without grade group upgrade (Table 5). We further analyse the correlations between the RAGE mRNA level and prostate cancer with the TCGA database. Statistical significant differences of the RAGE mRNA level were found between normal controls and prostate cancer patients (*p* < 0.0001, Figure 1A), and pathologic stage N1 and N0 patients (*p* = 0.0027, Figure 1C). However, no significant differences of the RAGE mRNA expression between pathologic N0 stage and N1 stage were observed (Figure 1B). The prostate cancer patients who posses higher RAGE expression were correlated with lower overall survival rate (Log Rank *p* = 0.025, Figure 1D).

**TABLE 3:** Distribution frequency of *RAGE* genotypes in 579 patients with prostate cancer with PSA ≤ 10

Variable	Grade group upgrade		AOR (95% CI)	*p* Value
	No (*n* = 156)	Yes (*n* = 114)		
rs1800625				
TT	133 (85.3%)	89 (78.1%)	1.00	
TC	21 (13.5%)	21 (18.4%)	1.611 (0.791–3.281)	0.189
CC	2 (1.2%)	4 (3.5%)	2.436 (0.412–14.401)	0.326
TC + CC	23 (14.7%)	25 (21.9%)	1.698 (0.868–3.321)	0.122
rs1800624				
TT	116 (74.4%)	84 (73.7%)	1.00	
TA	36 (23.1%)	26 (22.8%)	1.184 (0.632–2.221)	0.598
AA	4 (2.5%)	4 (3.5%)	1.339 (0.302–5.927)	0.701
TT + AA	40 (25.6%)	30 (26.3%)	1.202 (0.661–2.188)	0.546
rs2070600				
GG	85 (54.5%)	85 (74.6%)	1.00	
GA	64 (41.0%)	21 (18.4%)	0.304 (0.164–0.563)	<0.001*
AA	7 (4.5%)	8 (7.0%)	0.947 (0.313–2.862)	0.922
GA + AA	71 (45.5%)	29 (25.4%)	0.375 (0.214–0.657)	0.001*
rs184003				
GG	105 (67.3%)	82 (71.9%)	1.00	
GT	45 (28.8%)	28 (24.6%)	0.781 (0.434–1.403)	0.408
TT	6 (3.9%)	4 (3.5%)	0.750 (0.184–3.062)	0.688
GT + TT	51 (32.7%)	32 (28.1%)	0.777 (0.443–1.363)	0.379

Note

The odds ratios (ORs) and with their 95% confidence intervals (CIs) were estimated by logistic regression models.

* *p* Value < 0.05 as statistically significant.

**TABLE 4:** Odds ratio (OR) and 95% confidence interval (CI) of clinical status and *RAGE* rs1800625 genotypic frequencies in 579 patients with prostate cancer

Variable	Genotypic frequencies			
rs1800625	TT (*N* = 481)	TC + CC (*N* = 98)	OR (95% CI)	*p* Value
Pathologic Gleason grade group				
1 + 2 + 3	407 (84.6%)	77 (78.6%)	1.00	*p *= 0.141
4 + 5	74 (15.4%)	21 (21.4%)	1.500 (0.872–2.580)	
Clinical T stage				
1 + 2	419 (87.1%)	82 (83.7%)	1.00	*p *=0.364
3 + 4	62 (12.9%)	16 (16.3%)	1.319 (0.725–2.399)	
Pathologic T stage				
2	258 (53.6%)	48 (49.0%)	1.00	*p *= 0.400
3 + 4	223 (46.4%)	50 (51.0%)	1.205 (0.780–1.861)	
Pathologic N stage				
N0	441 (91.7%)	89 (90.8%)	1.00	*p *= 0.778
N1	40 (8.3%)	9 (9.2%)	1.115 (0.522–2.379)	
Seminal vesicle invasion				
No	381 (79.2%)	71 (72.4%)	1.00	*p *= 0.140
Yes	100 (20.8%)	27 (27.6%)	1.449 (0.883–2.377)	
Perineural invasion				
No	140 (29.1%)	15 (15.3%)	1.00	*p *= 0.005*
Yes	341 (70.9%)	83 (84.7%)	2.272 (1.267–4.074)	
Lymphovascular invasion				
No	403 (83.8%)	79 (80.6%)	1.00	*p *= 0.444
Yes	78 (16.2%)	19 (19.4%)	1.243 (0.712–2.168)	
D’Amico classification				
Low/intermediate risk	238 (49.5%)	42 (42.9%)	1.00	*p *= 0.232
High risk	243 (50.5%)	56 (57.1%)	1.306 (0.843–2.024)	
Biochemical recurrence				
No	340 (70.7%)	64 (65.3%)	1.00	*p *= 0.290
Yes	141 (29.3%)	34 (34.7%)	1.281 (0.809–2.029)	

Note

The ORs with analysed by their 95% CIs were estimated by logistic regression models.

^*^ *p* value < 0.05 as statistically significant.

**TABLE 5:** Odds ratio (OR) and 95% confidence interval (CI) of clinical status and *RAGE* rs1800625 genotypic frequencies in 343 patients with no grade group upgrade

Variable	Genotypic frequencies			
rs1800625	TT (*N* = 287)	TC + CC (*N* = 56)	OR (95% CI)	*p* Value
Pathologic Gleason grade group				
1 + 2 + 3	249 (86.8%)	46 (82.1%)	1.00	*p *= 0.362
4 + 5	38 (13.2%)	10 (17.9%)	1.424 (0.663–3.059)	
Clinical T stage				
1 + 2	239 (83.3%)	45 (80.4%)	1.00	*p *= 0.597
3 + 4	48 (16.7%)	11 (19.6%)	1.217 (0.587–2.522)	
Pathologic T stage				
2	165 (57.5%)	25 (44.6%)	1.00	*p *= 0.077
3 + 4	122 (42.5%)	31 (55.4%)	1.677 (0.942–2.985)	
Pathologic N stage				
N0	257 (89.5%)	50 (89.3%)	1.00	*p *= 0.953
N1	30 (10.5%)	6 (10.7%)	1.028 (0.407–2.599)	
Seminal vesicle invasion				
No	229 (79.8%)	38 (67.9%)	1.00	*p *= 0.058
Yes	58 (20.2%)	18(32.1%)	1.870 (0.996–3.513)	
Perineural invasion				
No	87 (30.3%)	8 (14.3%)	1.00	*p *= 0.014*
Yes	200 (69.7%)	48 (85.7%)	2.610 (1.185–5.749)	
Lymphovascular invasion				
No	238 (82.9%)	46 (82.1%)	1.00	*p *= 0.887
Yes	49 (17.1%)	10 (17.9%)	1.056 (0.499–2.235)	
D’Amico classification				
Low/intermediate risk	137 (47.7%)	22 (39.3%)	1.00	*p *= 0.246
High risk	150 (52.3%)	34 (60.7%)	1.412 (0.787–2.532)	
Biochemical recurrence				
No	204 (71.1%)	32 (57.1%)	1.00	*p *= 0.039*
Yes	83 (28.9%)	24 (42.9%)	1.843 (1.024–3.317)	

Note

The ORs with analysed by their 95% CIs were estimated by logistic regression models.

^*^ *p* value < 0.05 as statistically significant.

## 4 DISCUSSION

The correlations between the *RAGE* SNPs and prostate cancer were demonstrated in this study. The grouped Gleason score (GS) categories‐grade groups were proposed by Johns Hopkins Hospital in 2013 and adopted officially at the 2014 International Society of Urologic Pathology (ISUP) Consensus meeting.^43^, ^44^, ^45^ The grade group (GG) was defined as GS ≤ 6 (GG1), GS3 + 4 (GG2), GS4 + 3 (GG3), GS8 (GG4) and GS ≥ 9 (GG5), and each individual GG has a presumed similar prognosis for each GS category.^45^, ^46^ In our current study, most of the patients who developed GG upgrade were diagnosed as intermediate risk (45.3%) or high risk (48.7%) under D’Amico classification, suggesting a great proportion of GG2 to GG5 distribution to these patients with clinical T1 + T2 staging (91.9%) and pathologic N0 staging (94.5%) (Table 1).^47^, ^48^, ^49^, ^50^, ^51^, ^52^ We further examined the correlations between the *RAGE* SNPs and grade group upgrade of prostate cancer. We found that in prostate cancer patients with the *RAGE* SNPs rs2070600 ‘GA’ genotype were associated with lower risk to develop grade group upgrade (AOR = 0.628, 95% CI = 0.426–0.976; *p* = 0.019) (Table 2). Notably, we found that in 270 prostate cancer patients whose prostate‐specific antigen (PSA) ≤ 10, patients who carried the *RAGE* SNPs rs2070600 ‘GA’ genotype (AOR = 0.304, 95% CI = 0.164–0.563; *p* < 0.001) and ‘GA + AA’ polymorphic variants (AOR = 0.375, 95% CI = 0.214–0.657; *p* = 0.001) were associated with lower risk to develop grade group upgrade, respectively (Table 3).

The role of *RAGE* rs2070600 polymorphisms to cancer risk or disease susceptibility and prognosis remained controversial. Most studies have linked the *RAGE* rs2070600 polymorphic variant A allele with increased cancer risk and poor prognosis of disease,^16^, ^30^, ^42^, ^53^ However, in a study of lung cancer, RAGE was suggested to act as a tumour suppressor in lung cancer development, and the variant A allele of rs2070600 was suggested to be associated with decreased expression of the tumour suppressor gene *RAGE*.^53^ Although the role of RAGE in cancer development remained controversial, it was suggested that the *RAGE* rs2070600 polymorphisms were associated with the regulation of soluble RAGE (sRAGE) levels. In a study focused on Dutch population, the CC genotype of SNP rs2070600 (Gly82Ser) was found to be strongly associated with higher sRAGE levels.^54^ In gastric cancer, subjects who carried the rs2070600 AG genotype were observed to have a decreased ability to produce sRAGE.^30^ In lung cancer, the serum sRAGE level was found to be decreased during lung cancer progression and could reflect decreased RAGE expression in tissue, suggesting that the serum sRAGE may be a pivotal diagnostic biomarker for lung cancer.^55^ Compared with these results, although we lack of the data of sRAGE in our current study, it can be proposed that the *RAGE* rs2070600 polymorphic variant A allele might be linked with decreased level of sRAGE in prostate cancer, thereby decreasing the risk to develop grade group upgrade in prostate cancer patients, especially in those grade group upgrade patients whose PSA ≤ 10 (Tables 2 and 3).

We further examined the correlations between the *RAGE* SNPs and clinical status of prostate cancer. Intriguingly, we found that although the *RAGE* rs1800625 polymorphisms were not associated with the grade group upgrade of prostate cancer (Tables 2 and 3), however, the *RAGE* rs1800625 genotypic variants ‘TC + CC’ were found to be significantly associated with perineural invasion of prostate cancer (*p* = 0.005, Table 4). Moreover, in 343 prostate cancer patients with no grade group upgrade, the *RAGE* rs1800625 polymorphic variants ‘TC + CC’ were also found to be associated with perineural invasion (*p* = 0.014) and biochemical recurrence (*p* = 0.039) (Table 5). The *RAGE* rs1800625 polymorphisms were suggested to be associated with increased cancer risk in various cancers.^15^, ^17^, ^36^, ^56^, ^57^ Previous study has suggested that the C allele of rs1800625 may induce the expression of RAGE, and leads to chronic inflammatory conditions in diabetic retinopathy.^58^ Besides, the variant of the *RAGE* rs1800625 SNP was suggested to be associated with the hypomethylation of the promoter region of *RAGE* and contribute to the ulcerative colitis risk.^59^ Furthermore, after we analysed the TCGA database, we found that the RAGE mRNA level was significantly associated with prostate cancer tumorigenesis (Figure 1A) and pathologic N1 stage development (Figure 1C). The higher RAGE expression was also observed to be associated with lower overall survival rate in prostate cancer patients (Log Rank *p* = 0.025, Figure 1D). Moreover, Aboushousha et al. revealed that RAGE expression was significantly higher in prostate cancer lesions compared with prostatitis and benign prostatic hyperplasia.^60^ Taken together, it can be assumed that the *RAGE* rs1800625 polymorphic variants were associated with higher RAGE expression and tumour aggressiveness in prostate cancer development, leading to perineural invasion and biochemical recurrence in prostate cancer patients yet without grade group upgrade, and ultimately leads to poor prognosis and overall survival rate. However, future well‐designed studies are required to elucidate the exact mechanisms of *RAGE* SNPs in prostate cancer development, especially the influence of *RAGE* rs2070600 and rs1800625 SNPs to the sRAGE level regulation in prostate cancer tumour development and progression.

In conclusion, our results have demonstrated that the *RAGE* SNPs rs2070600 and rs1800625 were associated with prostate cancer grade group upgrade and tumour progression and prognosis. The prostate cancer patients who carried the *RAGE* rs2070600 allelic variant A allele were associated with lower risk to develop grade group upgrade, while the *RAGE* rs1800625 ‘TC + CC’ were associated with perineural invasion and biomedical recurrence in patients with no grade group upgrade. The *RAGE* rs1800625 might be linked with RAGE promoter hypomethylation and higher mRNA level in prostate cancer. The *RAGE* rs2070600 and rs1800625 polymorphisms may provide as pivotal markers to predict tumour aggressiveness, recurrence and prognosis in prostate cancer.

## CONFLICTS OF INTEREST

The authors declare that there is no conflict of interest.

## AUTHOR CONTRIBUTION

**Ying‐Erh Chou:** Conceptualization (equal); Writing‐original draft (equal); Writing‐review & editing (equal). **Ming‐Ju Hsieh:** Methodology (equal); Writing‐original draft (equal). **Shian‐Shiang Wang:** Resources (equal). **Chia‐Yen Lin:** Resources (equal). **Yen‐Yu Chen:** Methodology (equal). **Yung‐Chuan Ho:** Conceptualization (equal); Writing‐original draft (equal); Writing‐review & editing (equal). **Shun‐Fa Yang:** Conceptualization (equal); Writing‐original draft (equal); Writing‐review & editing (equal).

## Data Availability

The data used to support the findings of the present study are available from the corresponding author upon request.
